# Antibody Screening and Binding Prediction Analysis Targeting Stx2

**DOI:** 10.3390/antib15010011

**Published:** 2026-01-27

**Authors:** Jilei Wu, Chenghua Liu, Fenghao Peng, Zeyuan Yu, Chunxia Qiao, Guang Yang, Heng Luo, Keyi Sun, Ziyao Ning, Jing Wang, Yan Wen, Jijun Yu

**Affiliations:** 1 College of Biotechnology, Jiangsu University of Science and Technology, Zhenjiang 212000, China; 16631020721@163.com (J.W.); wander_999@163.com (H.L.); 13675264144@163.com (K.S.); 2 State Key Laboratory of National Security Specially Needed Medicines, Beijing 100850, China; lch0742@126.com (C.L.); pfh1744ss@163.com (F.P.); bioqcx@126.com (C.Q.); yangg62033@outlook.com (G.Y.); jingw_biomed@163.com (J.W.); 3 School of Artificial Intelligence and Data Science, University of Science and Technology of China, Hefei 230026, China; yuzeyuan@mail.ustc.edu.cn; 4 School of Pharmacy, Qingdao University, Qingdao 266071, China; 15176490679@163.com

**Keywords:** Shiga toxin type 2 (Stx2), human monoclonal antibody, ImmuneBuilder, structural prediction, *Escherichia coli* O157:H7 infection

## Abstract

**Background**: Shiga toxin (Stx), produced by enterohemorrhagic *Escherichia coli* (EHEC), is a highly potent exotoxin responsible for severe complications such as hemolytic uremic syndrome (HUS). Among its isoforms, Stx2 exhibits stronger cytotoxicity and poses greater clinical risk, yet no effective therapy currently exists. **Methods**: In this study, two human monoclonal antibodies, YG12-1 and YG12-2, were identified from a phage display library and systematically characterized using an integrated modeling–validation workflow. **Results**: Structural modeling with ImmuneBuilder and Rosetta revealed that YG12-2 possessed a longer CDRH3 topology, more short-range hydrogen bonds, and stronger electrostatic complementarity, corresponding to lower binding energy and higher apparent affinity in ELISA and SPR. Although YG12-2 had a better affinity, YG12-1 shows better protective activity in a murine model of acute peritoneal infection. This paradox highlights a non-linear relationship between structural affinity and biological efficacy, emphasizing the importance of functional epitope accessibility and pharmacokinetic behavior in determining neutralization outcomes. **Conclusions**: Overall, these results indicated that targeting Stx2 with YG12-1 and YG12-2 could serve as a promising protective strategy against *E. coli* O157:H7 infection.

## 1. Introduction

Shiga toxin (Stx), a major virulence factor secreted by enterohemorrhagic *Escherichia coli* (EHEC) O157:H7 and other related strains, exerts potent cytotoxic, enterotoxic, and neurotoxic effects [1,2,3,4,5]. Infection by Shiga toxin-producing *E. coli* (STEC) is a primary cause of hemolytic uremic syndrome (HUS), a life-threatening condition that frequently results in acute kidney injury in young children [6,7], especially those under five years of age [8,9,10]. The mortality rate of HUS ranges from 3% to 5% [7,11,12]. The development of HUS, hemorrhagic colitis (HC), and severe diarrheal illness in STEC-infected individuals is closely associated with the presence of Stx [13]. Stx is among the most lethal known bacterial exotoxins. Its toxicity has been estimated to be approximately 7000 times greater than that of the organophosphorus nerve agent VX and 45,000 times that of sarin [1,14,15]. Due to its extreme potency and ease of production, Stx is considered a potential biological warfare agent and a subject of considerable international concern. The Stx family comprises two major isoforms—Stx1 and Stx2—with Stx2 recognized as the primary virulence determinant in STEC-induced HUS [16]. Although the mechanisms of action of Stxs are thought to be the same, Stx2 is much stronger than Stx1 in mediating HUS [17]. The accumulation of Stx2 in renal microvascular endothelial cells is particularly damaging and is central to the pathogenesis of HUS [18,19,20].

Structurally, Stx is a member of the AB5 toxin family, consisting of a single enzymatically active A subunit and five identical B subunits responsible for cell binding. The B subunits bind to glycolipid receptors, mainly globotriaosylceramide (Gb3) [21] and globotetraosylceramide (Gb4) [22], which are abundantly expressed on endothelial cells in the kidneys and central nervous system. Following receptor-mediated endocytosis, the A subunit is translocated into the cytosol, where it inhibits protein synthesis by cleaving 28S rRNA, ultimately leading to cell death and organ damage [23,24].

Currently, there is no specific treatment for STEC infections. Clinical management remains largely supportive, emphasizing the maintenance of fluid and electrolyte balance [25,26]. In severe cases, renal replacement therapy such as dialysis may be required. The use of antibiotics is controversial, as several studies have indicated that they may exacerbate toxin release through bacterial lysis, potentially worsening the clinical outcome [27]. As a result, therapeutic strategies have increasingly focused on toxin neutralization as a means of mitigating host damage [8].

Among the various strategies developed to combat Stx-induced diseases, antibody-based therapies have shown particular promise. Monoclonal antibodies (mAbs), with their high specificity and affinity, represent the most widely adopted therapeutic format [28]. Both in vitro and animal model studies have demonstrated that mAbs can effectively neutralize Stx, prevent cytotoxicity, and mitigate the severity of hemolytic uremic syndrome (HUS) and its associated complications [29]. Their ability to target specific epitopes with minimal off-target effects makes them ideal candidates for therapeutic intervention [30].

Eculizumab, a monoclonal C5 inhibitor, is a therapeutic antibody approved for use during atypical HUS, which involves dysregulation of complement activation [31,32] although its use in STEC-induced HUS is not beneficial and is expensive [33,34]. There are three antibodies that have been tested in clinical trials: 1999 in Germany for Stx1 and 2 (polyclonal) [35], 2009 in the U.S. and Canada for Stx1b and Stx2a (chimeric) [36], and again in 2010 in Argentina for Stx2b (humanized) [37], but none have been approved by the FDA for use in humans [38].

**Figure 1:**
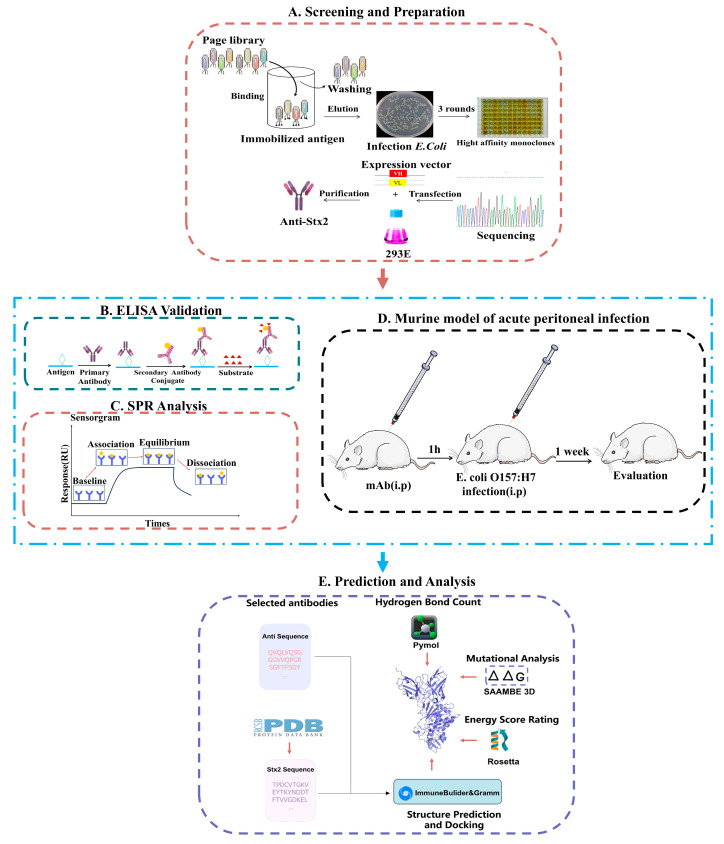
Schematic of antibody screening, prediction, and activity evaluation. (**A**) Screening and Preparation: Antibody clones were isolated by phage display panning, expressed in 293E cells, and purified for further analysis; (**B**,**C**) Binding activity of mAbs in vitro: (**B**) Binding affinity was assessed by ELISA. (**C**) Binding affinity was assessed by SPR. (**D**) Neutralization activity of mAbs in vivo. (**E**) Structural prediction and docking were performed using ImmuneBuilder (v1.1.1) and GRAMM (Web Sever); interface hydrogen-bond contacts were analyzed in PyMOL (v3.1), mutational effects (ΔΔG) were predicted using SAAMBE-3D (Web Sever), and interaction energies were finally calculated using Rosetta.

Here we describe the generation and characterization of two monoclonal antibodies designated YG12-1 and YG12-2 against Stx2 (Figure 1), which neutralize the cytotoxicity of Stx2. Targeting of Stx2 using the neutralizing antibodies generated herein may be an effective therapeutic strategy for the treatment of *E. coli* O157:H7 infection.

## 2. Results

### 2.1. Screening of Human Antibodies Targeting Stx2

To identify antibodies that bind Stx2 with high specificity and affinity, we employed a naïve human Fab phage display library for targeted screening.

**Figure 2:**
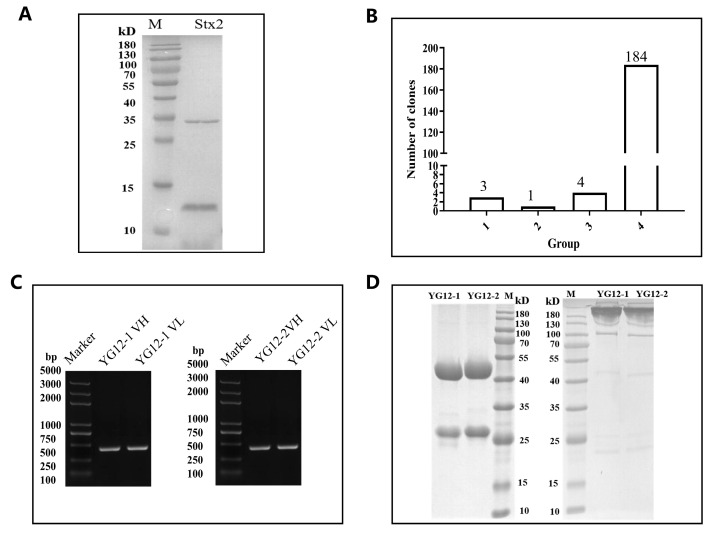
Characterization of Stx2 antibodies from a Naïve Human Fab Phage Library. (**A**) SDS-PAGE analysis of purified Stx2 protein under reducing conditions. (**B**) Screening of Stx2-binding clones by ELISA following three rounds of panning. A total of 192 clones were evaluated, and 8 clones showed specific binding to Stx2. Binding levels were categorized based on OD450 values: >1.5, 1.0–1.5, 0.5–1.0, and <0.5. (**C**) PCR amplification of VH and VL regions from clones YG12-1 and YG12-2. (**D**) Expression and purification of YG12-1 and YG12-2 antibodies analyzed by SDS-PAGE under reducing (left) and non-reducing (right) conditions.

Following three rounds of panning (Supplementary Table S1), 192 phage clones were selected and screened using ELISA. Eight clones, showing high reactivity (OD450 nm > 0.5), were subjected to DNA sequencing. Sequencing results revealed that four clones expressed only the heavy chain, while the remaining four clones had identical sequences (Figure 2A,B). Ultimately, two antibodies with specific binding to Stx2 were identified and designated YG12–1 and YG12–2 (see Table S4 for their VH and VL sequences).

The variable heavy (VH) and light (VL) chain genes of YG12–1 and YG12–2 were amplified by PCR (Figure 2C) and cloned into separate expression vectors. These constructs were co-transfected into HEK293E cells to produce full-length IgG antibodies. The antibodies were then purified from culture supernatants using Protein A affinity chromatography. SDS-PAGE analysis under both reducing and non-reducing conditions confirmed the purity and expected molecular weight of the antibodies (Figure 2D).

### 2.2. Evaluation of Anti-Stx2 Function Ex Vivo and In Vivo

**Figure 3:**
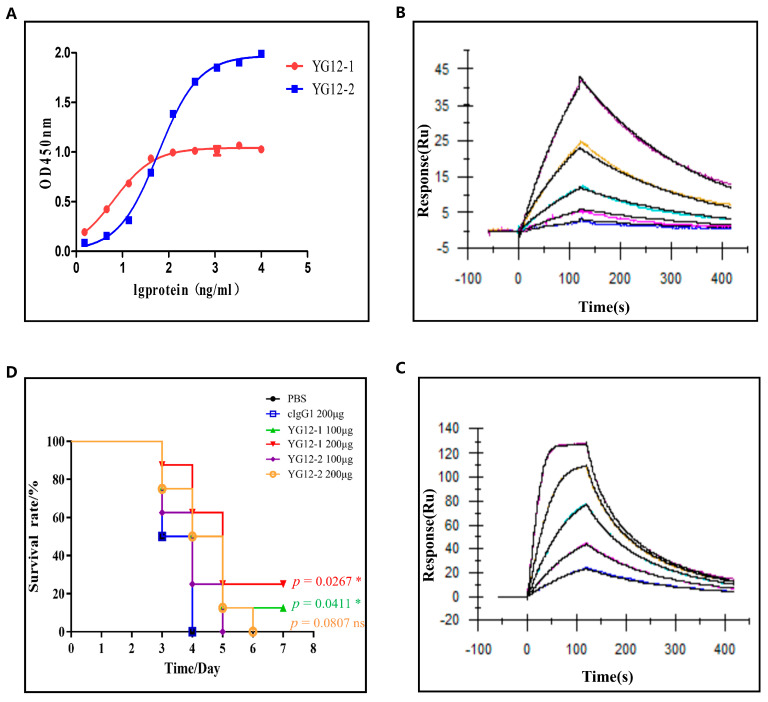
Evaluation of the activity of antibodies. (**A**) Binding affinity analysis of YG12 to Stx2; Stx2 protein was coated onto 96-well plates, and YG12 was added at various concentrations. Bound IgG was detected using horseradish peroxidase (HRP)-conjugated goat anti-human IgG. (**B**,**C**) Antibody affinity of YG12 to Stx2 was determined with surface plasmon resonance (SPR). SteadiCAP Protein A was immobilized on the surface of a CM5 chip, and YG12-1 (**B**) and YG12-2 (**C**) were captured by the immobilized SteadiCAP Protein A, respectively. Stx2protein was injected at the indicated concentrations. The data were analyzed using BlAcore T200 Evaluation software (V3.2). (**D**) Assessment of the protective effects of YG12 in vivo. Assessment of the protection activity of YG12 in a murine model of acute peritoneal infection. YG 12 or c-IgG was injected into the peritoneal cavity of C57bl/6J mice (*n* = 8) 1 h prior to challenge with *Escherichia coli* O157:H7 ATCC43895 (1 × 107 CFU/mouse). Data are presented as the percentage of mice surviving. Survival curves were determined using the Kaplan–Meier method and compared using the log-rank test, with *p*-values adjusted for multiple comparisons (Bonferroni correction) for the three experimental groups versus the PBS control. *: *p* < 0.05; ns: no significance.

The binding affinity of YG12 to Stx2 was subsequently evaluated by ELISA and surface plasmon resonance (SPR). The ELISA results showed that both YG12-1 and YG12-2 bind to Stx2 significantly, and the EC50 was 6.502 ± 1.12 ng/mL and 59.69 ± 1.99 ng/mL, respectively, (Figure 3A). SPR analysis revealed that YG12-1 and YG12-2 had binding affinities of 1.93 × 10^−7^ M and 2.42 × 10^−8^ M, respectively, (Figure 3B,C), and the YG12-2 exhibited superior binding affinity compared to YG12-1.

Next, to assess the in vivo protective efficacy of YG12 antibodies, we employed a murine model of acute peritoneal infection. YG12 antibodies or an IgG1 isotype control were administered intraperitoneally 1 h before a lethal challenge with *E. coli* O157:H7 ATCC43895. Survival was monitored over a 7-day period. As shown in Figure 3D, administration of YG12-1 resulted in significantly increased survival of mice in a dose-dependent manner, demonstrating that YG12-1 effectively protected mice from *E. coli* O157:H7 infection. Although YG12 prolonged the survival time, there was no statistical significance after adjustment.

These results indicate that both YG12-1 and YG12-2 exert protective effects against *E. coli* O157:H7 in vivo, with YG12-1 demonstrating superior efficacy. The experiment further underscores the therapeutic potential of YG12 antibodies in neutralizing Shiga toxin-producing bacterial infections.

### 2.3. Comparative Structural Analysis of YG12-1 and YG12-2 Antibodies

**Figure 4:**
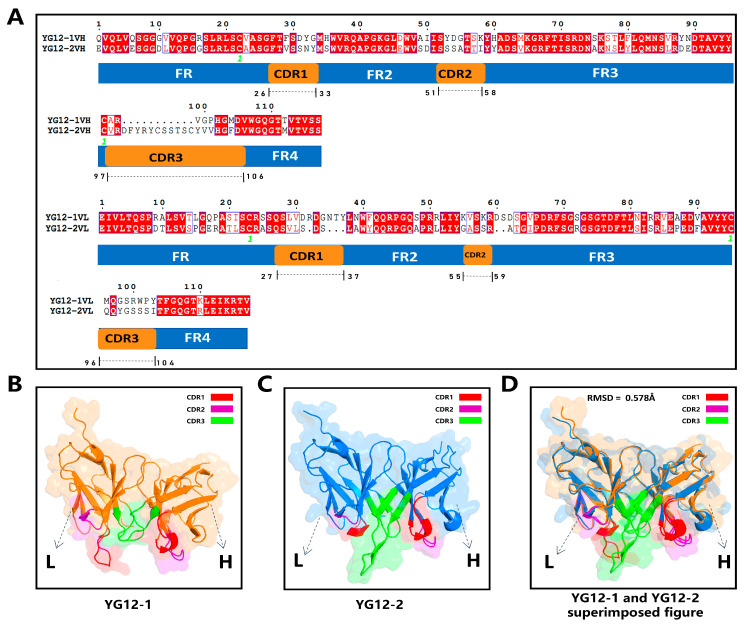
Sequence Alignment and Structural Analysis of YG12-1 and YG12-2 Antibodies. (**A**) Sequence alignment of VH and VL regions of YG12-1 and YG12-2, with annotated framework regions (FR1–FR4) and complementarity-determining regions (CDR1–CDR3); (**B**) Structural model of YG12-1 highlighting CDR1 (red), CDR2 (purple), CDR3 (green), and the framework (orange); (**C**) Structural model of YG12-2 with CDR1 (red), CDR2 (purple), CDR3 (green), and the framework (blue); (**D**) Superimposed structures of YG12-1 and YG12-2 showing an RMSD of 0.578 Å, indicating high overall structural similarity.

To compare the differences between the two antibodies, we analyzed the sequence and structural characteristics. Multiple sequence alignment (Figure 4A) revealed high conservation in the VH and VL framework regions of both antibodies, whereas the complementarity-determining regions (CDRs), particularly CDR3, exhibited significant differences. YG12-2 has a longer and more structurally distinct CDR3 region compared to YG12-1, suggesting broader epitope coverage and enhanced antigen complementarity.

ImmuneBuilder-based structural models (Figure 4B,C) further confirmed CDR3 differences, with YG12-2 exhibiting greater conformational deviation in the CDR3 loop. Structural superimposition analysis (Figure 4D) revealed a moderate RMSD of 0.578 Å, indicating conserved overall architecture but divergence in local regions critical for antigen interaction.

### 2.4. Prediction and Subsequent Validation of YG12-1 and YG12-2 Binding to Stx2 by Competitive ELISA

**Figure 5:**
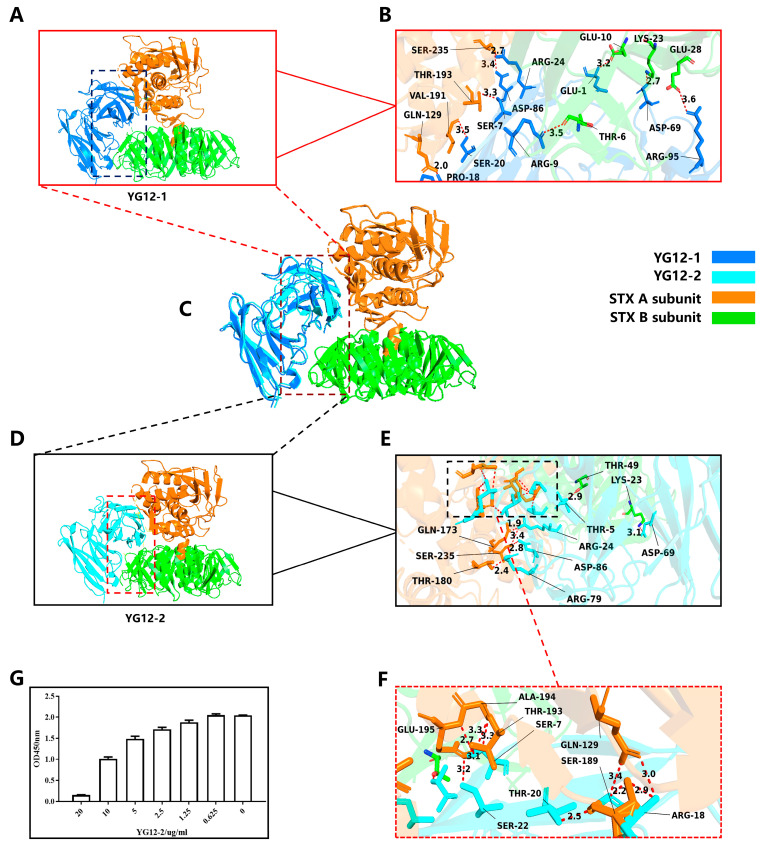
Structural Analysis of YG12-1 and YG12-2 Antibodies in Complex with Stx2 Antigen and Validation by Competitive ELISA. (**A**) Overall view of YG12-1: displays the overall hydrogen bond distribution of the antibody; (**B**) Detailed view of YG12-1 binding interface: focuses on the hydrogen bond interactions between the antibody and the antigen contact surface; (**C**) Superimposed image of YG12-1 and YG12-2: shows the overlapping binding sites between YG12-1 and YG12-2 antibodies; (**D**) Overall view of YG12-2: displays the overall hydrogen bond distribution of the antibody; (**E**) Detailed view of YG12-2 binding interface: focuses on the hydrogen bond interactions between the antibody and the antigen contact surface; (**F**) Detailed view of the YG12-2 binding interface: enlarged view of the boxed region in panel E. (**G**) YG12-1 and YG12-2 bound to the same region of Stx: YG12-1 and YG12-2 competed with each other in a concentration-dependent manner. YG12-1 was coated onto 96-well plates, and YG12-2 was added at various concentrations after pre-incubated with His-Stx2. Bound His-Stx2 was detected using an anti-His antibody.

To further elucidate the antibody recognition mechanism, we conducted a detailed analysis of the predicted antibody–antigen complexes of YG12-1 and YG12-2 (Figure 5).

In terms of hydrogen bond distribution, YG12-2 had 16 hydrogen bonds, significantly more than YG12-1. Moreover, the distance of YG12-2 was shorter, which greatly enhanced the binding stability (See Figure 5A–F, Supplementary Table S2).

Additionally, YG12-2 forms multiple hydrogen bonds at key binding sites, establishing a multi-point contact spatial conformation that further stabilizes the antibody–antigen binding. These multi-point contact binding sites (GLN129, GLN173, SER189, GLU195, and SER235) provide stronger affinity and binding stability (see Figure 5E,F), allowing YG12-2 to exhibit superior stability and affinity during antigen recognition. In conclusion, YG12-2 exhibits stronger binding effects and stability through more short hydrogen bonds and multi-point contact binding sites, demonstrating enhanced affinity and binding stability, especially through the synergistic effect of hydrogen bonds [39].

Both the structural visualization (Figure 5C) and the epitope mapping results (Supplementary Table S2) demonstrate that YG12-1 and YG12-2 occupy similar binding regions on Stx2. They share four common epitope residues (LYS23, GLN129, THR193, and SER235), confirming that the two antibodies recognize partially overlapping epitopes, which may account for their competitive binding behavior.

To validate whether YG12-1 and YG12-2 recognize overlapping epitopes on Stx2, we performed a competitive ELISA (Figure 5G). As the concentration of YG12-2 increased, a gradual reduction in OD450 nm signal was observed, indicating that YG12-2 effectively inhibited the binding of Stx2 to YG12-1 in a concentration-dependent manner. These results indicate that YG12-1 and YG12-2 target partially overlapping epitopes on the Stx2 molecule, suggesting potential competition at the antigen-binding interface. This experimental result validates our previous complex structure analysis and further supports the hypothesis that YG12-1 and YG12-2 recognize partially overlapping epitopes.

### 2.5. Validation of Docking-Predicted Epitopes by Mutational Analysis

To validate the accuracy of the epitopes predicted by molecular docking, we performed an epitope-directed mutational analysis using SAAMBE-3D (Web Sever). As shown in Supplementary Table S5, all simulated mutations resulted in positive ΔΔG values, indicating destabilizing effects on antigen–antibody binding. This observation is consistent with the overall trend reported in the SKEMPI v2.0 database, in which the majority of single-point mutations are destabilizing, reflecting the evolutionary optimization of native protein–protein interfaces.

Statistical analysis revealed significant differences between the two groups for both antibodies (Supplementary Table S6). Specifically, the comparison between the negative and positive control groups yielded a t-statistic of −2.336 (*p* = 0.0435) for YG12-1 and −3.109 (*p* = 0.0106) for YG12-2, both reaching statistical significance (*p* < 0.05). These results demonstrate that mutations at the predicted binding epitopes disrupt antigen–antibody interactions more strongly than mutations at non-binding sites, providing statistical support for the functional importance of the docking-predicted epitopes.

**Figure 6:**
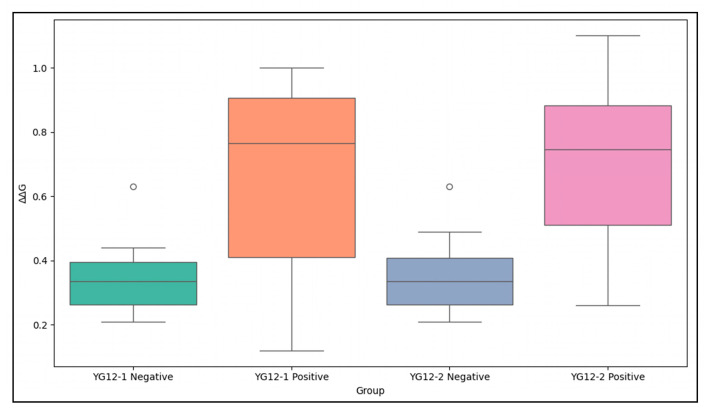
Comparison of ∆∆G Values for YG12-1 and YG12-2 Negative and Positive Control Groups. The negative control groups (non-binding epitopes) exhibit lower ∆∆G values compared to the positive control groups (binding epitopes), which show significantly higher ∆∆G values. The data highlight the distinct impact of mutations in the binding and non-binding regions on antigen–antibody interaction stability. A detailed ∆∆G table is provided in Supplementary Table S5.

In terms of the numerical analysis (Figure 6), the ∆∆G values of the negative control group were generally lower (YG12-1: 0.36 ± 0.12 kcal/mol; YG12-2: 0.37 ± 0.13 kcal/mol), indicating that mutations at these non-binding sites had minimal impact on antigen–antibody binding stability. In contrast, the ∆∆G values of the positive control group were significantly higher (YG12-1: 0.65 ± 0.31 kcal/mol; YG12-2: 0.70 ± 0.25 kcal/mol), further supporting their role as key residues at the antigen–antibody interface.

Further analysis showed that several overlapping epitopes predicted for YG12-1 and YG12-2, including LYS23, GLN129, and THR193, consistently exhibited elevated ΔΔG values in both antibodies. These results indicate that these residues make important functional contributions to the binding interfaces of both antibodies. In comparison, mutation of SER235 resulted in lower ΔΔG values (YG12-1: 0.26 kcal/mol; YG12-2: 0.45 kcal/mol), suggesting that SER235 has a supportive or stabilizing role in antigen–antibody interactions rather than being a primary binding determinant.

Consistent with the competitive ELISA results, the mutational analysis further indicates that YG12-1 and YG12-2 recognize partially overlapping epitopes. Overall, the epitopes predicted by molecular docking play a key role in antigen–antibody interactions, further validating the accuracy of the docking results.

### 2.6. Energy Scoring of YG12-1 and YG12-2 Antigen–Antibody Complexes

To quantitatively assess the thermodynamic stability of antigen–antibody complexes formed by YG12-1 and YG12-2, we employed the Rosetta Energy score module to calculate the binding energies of the structures (Supplementary Table S3). This analysis complemented our predictive studies and offered a mechanistic understanding of the disparities in binding affinity and conformational adaptability.

For the YG12-1-Stx2 complex, after energy prediction, the binding energy was −1993.9488 kcal/mol. The binding energy of the YG12-2-Stx complex was −2054.32778 kcal/mol, indicating that YG12-2 had a lower binding energy and better stability.

These results were consistent with previous analyses of hydrogen bond density and spatial conformation. Overall, these findings confirmed that YG12-2 had superior thermodynamic stability and binding performance compared to YG12-1. This energy-based evaluation further supports the structural advantages of YG12-2 and provides a foundation for further investigation in functional and therapeutic development.

## 3. Discussion

Over the past three decades, substantial efforts have been devoted to developing neutralizing antibodies against Shiga toxins (Stxs). Several monoclonal and recombinant antibodies, including urtoxazumab (TMA-15) and Shigamabs^®^ (caStx1/caStx2), demonstrated promising neutralizing activity in vitro and favorable safety profiles in early clinical studies, but none have been approved by the FDA for use in humans.

In this study, we generated two novel human mAbs specifically targeting Stx2, YG12-1 and YG12-2. YG12-2 had higher affinity measured by ELISA and SPR. Compared to YG12-2, YG12-1 exhibited a higher equilibrium constant (KD), coupled with a slower association rate constant, indicating a slower binding rate and suggesting potentially reduced complex stability. Corresponding structural analysis suggested that YG12-1 possesses a shorter CDR3 region and fewer hydrogen-bond networks at the binding interface.

Despite its higher structural affinity and predicted thermodynamic stability, YG12-2 conferred weaker protection in the murine model of *E. coli* O157:H7 infection. This observation highlights a non-linear relationship between in vitro binding affinity and in vivo neutralization efficacy, a phenomenon that has been reported for other toxin-neutralizing antibodies [40]. We hypothesized that YG12-1 may target a more critical functional epitope involved in toxin binding or intracellular trafficking [41], whereas the extended CDRH3 of YG12-2, although energetically favorable, could impose steric constraints that reduce effective antigen accessibility [42]. The differences in pharmacokinetic properties, such as serum stability or Fc-mediated clearance, lead to the different effects in vivo [43]. However, these interpretations were speculative, as such parameters were not directly assessed in the present study.

Further research will employ more detailed epitope localization methods (such as site-directed mutagenesis or cryo-electron microscopy techniques) to assess the sub-epitope criticality and accessibility, together with pharmacokinetic profiling in animal models (including half-life, biodistribution, and clearance), which will be necessary to clarify their respective contributions in vivo.

Collectively, our findings demonstrated that integrating computational structure-based predictions with functional validation could provide a more comprehensive framework for assessing therapeutic antibody candidates. The framework in this study provided a scalable strategy for early-stage antibody evaluation and delineated the boundaries of structure-based prediction. However, this work also has certain limitations, as we only performed a preliminary, computation-based simulation of the binding epitopes for the two antibodies. In the future, we will carry out more efforts to obtain a high-resolution three-dimensional structure and reveal conformational changes to enable a rational transition from structural affinity optimization to functional potency optimization.

## 4. Conclusions

In this study, we generated two novel anti-Stx2 human mAbs, YG12-1 and YG12-2; we further analyzed the binding characteristics of the two antibodies against Stx2, and these antibodies demonstrated certain neutralizing activity, providing new therapeutic potential for Shiga toxin-producing bacterial infections.

### 4.1. Experimental Procedures

All experiments in this study were performed from August 2024 to August 2025 under the approved protocol IACUC-DWZX-2025-728.

### 4.2. Cell Lines and Reagents

HEK293F cells (Thermo Fisher Scientific, Waltham, MA, USA) were cultured in FreeStyle™ 293 expression medium (Gibco, Grand Island, NY, USA; Thermo Fisher Scientific) supplemented with 2 mM L-glutamine (Gibco, Thermo Fisher Scientific Inc.). The cells were maintained in a humidified atmosphere with 8% (*v*/*v*) CO_2_ at 37 °C. The cultures were agitated at 125 rpm on an orbital shaker.

### 4.3. Selection of Hu-Anti-Stx2

Hu-anti-Stx2 was isolated from a phage display antibody library using the standard procedure. In brief, Stx2 was coated on Nunc-immunotube (Thermo Scientific Nunc^®^, 444202) at 4 °C overnight. Then phages (1012 plaque-forming units [PFU]/mL) were incubated with Stx2 for 1 h, and unbound phages were removed. The bound phages were eluted using 0.1 M Gly-HCl (pH 2.2). *E. coli* TG1 cells were infected with the eluted phages. The amplified phages were then subjected to the next round of panning. After three rounds of panning (Supplementary Table S1), single colonies were randomly selected and further screened by ELISA. The sequences of the specific phage clones binding to Stx2 were analyzed.

### 4.4. Plasmid Construction and Anti-Stx2 Expression

The genes encoding VH and VL were amplified by PCR from positive clone, respectively, and then cloned into a modified eukaryotic expression vector pcDNA3.0, respectively, and co-transfected into HEK293F cells. Monoclonal antibodies from the culture supernatants were purified by the Protein A affinity resin.

### 4.5. Binding Affinity of Hu-Anti-Stx2 Antibody

Binding affinity of Stx2 antibody to Stx2 was determined by ELISA. Briefly, 96-well plates were coated with Stx2 or its subunits individually overnight at 4 °C. After blocking, serially diluted antibodies (YG12-1/YG12-2) were added to wells and incubated at 37 °C for 1 h. The horseradish peroxidase goat anti-human antibody (1:40,000; Jackson, 109-035-003) was used to detect the bound antibody.

The Anti-Stx2 binding kinetics were analyzed using a Biacore T200 instrument. SteadiCAP Protein A was immobilized on the surface of BIAcore’s CM5 sensor chip by a standard coupling protocol. The antibody was injected over the chip surface at a flow rate of 30 µL/min. Stx2 (8–128 nM in 2-fold serial dilutions) were sequentially injected and incubated with anti-Stx2 bound on the tips. The Biacore T200 Evaluation software was used to analyze data.

### 4.6. Competitive ELISA

The 96-well plates were coated with YG12-1 overnight. After blocking with 5% non-fat milk at 37 °C for 1 h, serial dilutions of YG12-2 were added to the wells, respectively, which mixed with 10 μg/mL His-Stx2; after being incubated at 37 °C for 1 h and washed 5 times with PBST, the monoclonal mouse Anti-His antibodies (TransGen HT501-01) were added for 45 min at 37 °C. Followed by the addition of a HRP-conjugated goat anti-mouse secondary antibody for 30 min at 37 °C, the plates were washed 3 times and incubated for 30 min with TMB. Optical density at 450 nm was measured in a microtiter plate reader.

### 4.7. Acute Peritoneal Infection Murine Model

Female 6–8-week-old C57BL/6J mice (8 mice/group in each experiment) were purchased from Vital River Company, Beijing, China (SCXK2021-0006) and housed under specific pathogen-free (SPF) conditions in filter-top cages. A total of 5 experimental groups were used in the study, consisting of 40 mice in total (8 mice per group). The animal study was reviewed and approved by the Institutional Animal Care and Use Committee of the Academy of Military Medical Sciences (IACUC-DWZX-2025-728), following ARRIVE guidelines. C57BL/6J mice were injected intraperitoneally with YG12 (100 μg/mouse or 200 μg/mouse) at 1 h before the bacterial challenge, then animals were challenged intraperitoneally with *Escherichia coli* O157:H7 ATCC43895 (1 × 10^7^ CFU/mouse). Survival rate was assessed for 1 week after bacterial challenge. All of the mice were euthanized by CO_2_ inhalation after the experiment. Statistical analysis was performed by analysis of survival curves by the log-rank (Mantel–Cox) test with *p*-values adjusted for multiple comparisons (Bonferroni correction) for the three experimental groups versus the PBS control using Prism 6 (GraphPad).

### 4.8. Multiple Sequence Alignment

The amino acid sequences of the variable heavy (VH) and variable light (VL) chains of YG12-1 and YG12-2 were aligned using the ESPript 3.0 tool [44]. The sequence comparison primarily focused on identifying variations within the complementarity-determining regions (CDRs), with particular emphasis on CDR3. The alignment results were exported from the ESPript 3.0 server and analyzed to assess sequence conservation and potential structural implications.

### 4.9. Antibody Structure Prediction

To investigate the spatial conformation of antibodies YG12-1 and YG12-2, we employed the latest version of ImmuneBuilder (v1.1.1) and integrated deep learning techniques for structure prediction [45]. We input the full-length amino acid sequences of YG12-1 and YG12-2 into ImmuneBuilder to generate 3D antibody structures. The predicted models accurately capture the classic features of antibodies, including the folding of the light and heavy chains, complementarity-determining regions (CDRs), and the topology of antigen-binding.

To ensure the model’s reliability and high resolution, ImmuneBuilder utilizes a deep learning architecture similar to AlphaFold, adopting an ensemble method for structure prediction and selecting the optimal structure by minimizing the root mean square deviation (RMSD) of the α and β chains. The model successfully captures molecular interactions across multiple spatial scales, laying a solid foundation for subsequent antigen docking and interaction analyses [45].

Compared to AlphaFold, ImmuneBuilder’s prediction speed is approximately 250 times faster, enabling large-scale antibody structure predictions. The model efficiently processes vast amounts of data, predicting over 1.5 million TCR structures, significantly increasing the available structural data, and providing strong support for antibody-related research and applications.

### 4.10. Antigen–Antibody Docking Analysis

The crystal structure of Shiga toxin (PDB ID: 1DMO), including both the enzymatic A subunit and receptor-binding B subunit, was obtained from the RCSB PDB database for docking simulations. The structures of the YG12-1 and YG12-2 antibodies were predicted using ImmuneBuilder (v1.1.1), and these were subsequently combined with the Gramm(Web Sever) docking method to simulate the interactions between the antibodies and Stx2. Two distinct docking models were generated using Gramm: one for YG12-1 bound to full-length Stx2, and another for YG12-2 bound to full-length Stx2. These models enabled us to compare the binding modes of the antibodies with the toxin.

The Gramm docking method employs a multi-step computational process to predict protein–protein docking [46]. Initially, a global search is performed to generate a broad set of potential docking configurations by sampling the relative orientations and positions of the antibody and Stx2. Following this, local optimization is carried out to refine these configurations, adjusting the positions and orientations of the proteins to identify the most stable docking mode with the lowest energy. The docking results are evaluated using a scoring function that incorporates factors such as van der Waals interactions, electrostatic interactions, and solvation effects, ultimately selecting the most stable binding mode.

In this study, the GRAMM docking method was used to generate five docking models for each antibody (YG12-1 and YG12-2) in complex with Shiga toxin 2 (Stx2), yielding a total of ten docking models. The docking results were evaluated based on energy scores, geometric complementarity, and the involvement of complementarity-determining regions (CDRs) at the binding interface. Models exhibiting the lowest predicted energy, optimal geometric fit, and the strongest CDR–antigen complementarity were considered the most plausible binding conformations. For subsequent analyses, the top-ranked docking model for YG12-1–Stx2 and YG12-2–Stx2 was selected, as these models represent the most stable configurations predicted by GRAMM.

By combining the antibody structures predicted by ImmuneBuilder with the Gramm docking method, we were able to accurately capture the interactions between the antibodies and Stx2, providing theoretical support for subsequent affinity optimization, energy scoring, and antibody optimization design.

### 4.11. Binding Energy Scoring

To quantify binding stability, we employed the Rosetta energy scoring framework, which evaluates thermodynamic stability based on a composite of van der Waals interactions, electrostatics, hydrogen bonding, and solvation energies. Each of the two complexes predicted by ImmuneBuilder & Gramm was converted to PDB format and evaluated using Rosetta’s Energy_score module [47].

For each antibody–antigen complex, three independent binding energy calculations were performed, and the average value was used for subsequent analyses to minimize stochastic variability. Lower binding energy values indicated tighter and more stable interactions at the antibody–antigen interface. Energy distribution analyses were further integrated with structural metrics to identify key contributing residues and interface compactness.

Rosetta scoring results were consistent with CDR architecture and interface complementarity, supporting the reliability of ImmuneBuilder & Gramm-based structural predictions. These results provide a thermodynamic framework for affinity maturation and antibody design based on structure–function correlation.

### 4.12. Hydrogen Bond Analysis

Hydrogen bonding is a major stabilizing force in protein–protein interactions, particularly at antibody–antigen interfaces. To further characterize interfacial interactions, we used PyMOL (v3.1) [48] to analyze hydrogen bonds in the two docking models. This included quantification of bond numbers, spatial locations, and involvement of critical residues. Hydrogen bonds were defined based on a uniform geometric criterion, with an interatomic distance cutoff of ≤3.5 Å, which is commonly applied in structural analyses of antibody–antigen complexes [49].

The hydrogen bond network was visualized and annotated in 3D to identify dominant polar contacts contributing to complex stabilization. Comparative analyses revealed differences in hydrogen bonding patterns across complexes, correlating with predicted binding affinities.

### 4.13. SAAMBE-3D-Based ΔΔG Prediction for Epitope Mutations in YG12 Antibodies

To validate the accuracy of epitope predictions from molecular docking, we employed SAAMBE-3D [50], a machine learning-based tool that predicts changes in binding free energy (∆∆G) due to single amino acid mutations in antibody–antigen complexes. SAAMBE-3D uses 33 knowledge-based features, including amino acid hydrophobicity, hydrophilicity, volume, and protein structural properties, to compute ∆∆G values (in kcal/mol). Positive ∆∆G values indicate destabilizing mutations that reduce binding affinity, while negative values suggest stabilizing mutations that enhance it.

For epitope mutation analysis, we established negative and positive control groups based on docking-predicted epitopes. In the negative control, we mutated non-binding residues outside the predicted epitopes—located on the antigen surface but uninvolved in interactions—to avoid significant impacts on complex stability. In the positive control, we mutated all predicted binding epitopes for YG12-1 and YG12-2, as these key regions are expected to influence binding affinity, providing a reference for validation.

To simulate mutation effects while minimizing surface conformational changes, we substituted selected residues with alanine (A), eliminating side-chain influences and isolating mutation-specific ∆∆G effects.

All ∆∆G predictions were conducted via the SAAMBE-3D web server. Statistical comparisons of average ∆∆G values for overlapping epitopes between YG12-1 and YG12-2 used paired *t*-tests, with *p* < 0.05 considered significant. Data visualizations and analyses were generated in Python (V3.9) and uploaded to Figshare.

### 4.14. Data Availability

The data are released under a CC-BY license. This repository contains the modeled antibody–antigen complex structures in PDB format and the raw numerical data underlying all figures and statistical analyses. Below is the list of our files.

2.1 Screening of Human Antibodies Targeting Stx2:

Figure 2: Characterization of Stx2 Antibodies from a Naïve Human Fab Phage Library

- A: SDS-PAGE analysis of purified Stx2 protein.
  File: “Figure 2A SDS-PAGE of Stx2 Protein.png”
- B: ELISA screening of Stx2-binding clones after panning.
  File: “Figure 2B ELISA Screening.png”
- C: PCR amplification of VH and VL regions from YG12-1 and YG12-2.
  File: “Figure 2C PCR Amplification.png”
- D: SDS-PAGE analysis of YG12-1 and YG12-2 antibodies.
  File: “Figure 2D SDS-PAGE of YG12 Antibodies.png”

2.2 Binding Affinity Evaluation and In Vivo Neutralization Ability Assessment of YG12 Antibodies

Figure 3: Evaluation of the Activity of YG12 Antibodies

- A: Binding affinity analysis of YG12 to Stx2 using ELISA.
  File: “Figure 3A Original Figure of In Vivo Protective Activity of YG12.png”
- B & D: SPR analysis of YG12-1 (B) and YG12-2 (D) binding to Stx2.
  File: “Figure 3B Raw SPR Sensorgrams for YG12-1 Binding Analysis.png”
  File: “Figure 3D Raw SPR Sensorgrams for YG12-1 Binding Analysis.png”
- C: In vivo protection assessment of YG12 in a murine model.
  File: “Figure 3C Original Figure of YG12 Binding Affinity to Stx2.png”

2.3 Comparative Structural Analysis of YG12-1 and YG12-2 Antibodies

Figure 4: Sequence Alignment and Structural Analysis of YG12-1 and YG12-2 Antibodies

- A: Sequence alignment of VH and VL regions of YG12-1 and YG12-2, showing framework regions (FR1–FR4) and complementarity-determining regions (CDR1–CDR3).
  File: “Figure 4A Original Image Alignment of the VL Chain of YG Antibody and Secondary Structure of YG12-1VL.png”
  File: “Figure 4A Original Image Alignment of the VL Chain of YG Antibody and Secondary Structure of YG12-2VL.png”
  File: “Figure 4 Sequence Alignment of the VH Region of YG Antibody.fas”
  File: “Figure 4 Sequence Alignment of the VL Region of YG Antibody.fas”
- B: Structural model of YG12-1
  File: “Figure 4B lm-YG12-1 Antibody.pdb”
- C: Structural model of YG12-2
  File: “Figure 4C lm-YG12-2 Antibody.pdb”

2.4 Prediction and Subsequent Validation of YG12-1 and YG12-2 Binding to Stx2 by Competitive ELISA

Figure 5: Structural and Binding Analysis of YG12-1 and YG12-2 Antibodies

- A&C&D: Superimposed structures of YG12-1 and YG12-2 with STX2.
  File: “Figure 5A,C,D YG12-1 & YG12-2 Superimposed with STX2 Diagram.pse”
- B: The position where lm_YG12-1 binds to the antibody, marked with 9 hydrogen bonds.
  File: “Figure 5B The Position Where lm_YG12-1 Binds to the Antibody is Marked (9 Hydrogen Bonds).pse”
- E&G: The binding position of lm_YG12-2 with the antibody, marked with 16 hydrogen bonds.
  File: “Figure 5E,G The Binding Position of lm_YG12-2 with the Antibody is Marked (16 Hydrogen Bonds).pse”
- F: Original competitive ELISA figure for YG12-1 and YG12-2.
  File: “Figure 5F Original Competitive ELISA Figure for YG12-1 and YG12-2.png”
- Original docking results
  File: “Figure 5. GRAMM Docking Results for YG12-1”
  File: “Figure 5. GRAMM Docking Results for YG12-2”

2.5 Validation of Docking-Predicted Epitopes by Mutational Analysis

Figure 6. Comparison of ∆∆G Values for YG12-1 and YG12-2 Negative and Positive Control Groups

- A: This file contains the results from the epitope mutation analysis based on the SAAMBE tool.
  File: “Figure 6. Epitope Analysis Results Based on SAAMBE.txt”
  Each file is clearly labeled to correspond with the relevant content in the manuscript.

All other data generated or analyzed during this study are included within the article and its Supplementary Materials. No datasets are subject to embargo or access restrictions.

## Data Availability

The original data presented in the study are openly available in FigShare at https://doi.org/10.6084/m9.figshare.30397450 [51], assessed on 25 November 2025.

## Supplementary Materials

The following supporting information can be downloaded at https://www.mdpi.com/article/10.3390/antib15010011/s1, Table S1: Three-round selection of Hu-Anti-Stx2; Table S2: Binding Epitopes of YG12-1 and YG12-2 with Stx; Table S3: Comparison of the binding energies of YG12-1 and YG12-2 to Stx2; Table S4: Full-length VH and VL sequences of YG12-1 and YG12-2 antibodies; Table S5: ΔΔG Prediction for Mutations in YG12-1 and YG12-2; Table S6: *t*-Test Results for ∆∆G Differences Between Negative and Positive Control Groups (YG12-1 and YG12-2).
