# Supplementary material for: Antibody Screening and Binding Prediction Analysis Targeting Stx2
Source: Antibodies (Basel). 2026 Jan 27;15(1):11. doi: 10.3390/antib15010011 (PMC12921925; doi:10.3390/antib15010011)
Supplement: Supplementary file 1 [file antibodies-15-00011-s001.zip › antibodies-4038333-supplementary.pdf]

Table S1. Three-round selection of Hu-Anti-Stx2.

	Amount of Stx2 Protein (μg)	Input	Output
1st round	10	1.2×10^{12}	1.1×10^{4}
2nd round	10	2.5×10^{12}	1.2×10^{5}
3rd round	10	3.2×10^{12}	6.2×10^{6}

Table S2. Binding epitopes of YG12-1 and YG12-2 with Stx.

ID	Antibody Binding Site	Corresponding Antigen Epitope	Hydrogen Bond Distance(Å)
YG12-1-Stx	PRO-18	GLN-129	2
	ASP-69	LYS-23	2.7
	ARG-24	SER-235	2.7
	GLU-1	GLU-10	3.2
	SER-7	THR-193	3.3
	ASP-86	SER-235	3.4
	ARG-9	THR-6	3.5
	SER-20	VAL-191	3.5
	ARG-95	GLU-28	3.6
YG12-2-Stx	ARG-24	GLN-173	1.9
	ARG-18	SER-189	2.2
	ARG-79	THR-180	2.4
	THR-20	SER-189	2.5
	SER-7	GLU-195	2.7
	ASP-86	SER-235	2.8
	THR-5	THR-49	2.9
	ARG-18	SER-189	2.9
	ARG-18	GLN-129	3
	SER-7	THR-193	3.1
	SER-22	GLU-195	3.2
	SER-7	ALA-194	3.3
	SER-7	ALA-194	3.3
	ASP-69	LYS-23	3.4
	ARG-18	GLN-129	3.4
	ARG-24	SER-235	3.4

Table S3. Comparison of the binding energies of YG12-1 and YG12-2 to Stx2.

ID	Energy Score
YG12-1_Stx2	−1993.94876
YG12-2_Stx2	−2054.32778

Abbreviations: Stx2, Shiga toxin type 2.

 https://doi.org/10.3390/xxxxx

Table S4. Full-length VH and VL sequences of YG12-1 and YG12-2 antibodies.

ID	VH	VL
YG12-1	QVQLVQSGGGVVQPGRSLRLSCVASGFTFSDYG MHWVRQAPGKGLDWVAIISYDGTSK-YHADSMKGRFTISRD-NSKSTLFLQMNSVRYNDTAVYYCARVGPHGM DVWGQGTTVTVSS	EIVLTQSPRALSVTLGQPASIS-CRSSQSLVDRDGNTYLNWFQQRPGQSPRRLI-YKVSKRDSDSGVPDRFSGSGSGTDFTLNIRRVEA EDVAVYYCMQGSRWPYTFGQGTKLEIK
YG12-2	EVQLVESGGDLVQPGGSLRLS-CAASGFTVSSNYMSWVR-QAPGKGLEWVSDISSSATTIY-YADSVKGRFTISRDNAKNSLYLQMNSLRDEDTA VYYCVRDFYRYCSST-SCYVVHGFDVWGQGTMVTVSS	EIVLTQSPDTLSVSPGER-ATLSCRASQSVLSDSLAWYQQRPGQAPRLLI-YGASSRATGIP-DRFSGRGSGTDFTLSISRLEPEDFAVYYCQQYGSS SITFGQGTRLEIK

Table S5. ΔΔG prediction for mutations in YG12-1 and YG12-2.

ID	Chain	Position	Original AA	Mutated AA	ΔΔG Prediction (kcal/mol)	Stability Effect
			negative control			
YG12-1	A	52	V	A	0.35	Destabilizing
	A	61	E	A	0.28	Destabilizing
	A	91	R	A	0.44	Destabilizing
	E	25	G	A	0.44	Destabilizing
	E	31	T	A	0.21	Destabilizing
	E	55	N	A	0.63	Destabilizing
	F	5	V	A	0.32	Destabilizing
	F	31	T	A	0.21	Destabilizing
	F	60	G	A	0.38	Destabilizing
			positive control			
	A	129	Q	A	0.89	Destabilizing
	A	193	T	A	0.86	Destabilizing
	A	235	S	A	0.26	Destabilizing
	A	191	V	A	1.00	Destabilizing
	E	6	T	A	0.67	Destabilizing
	F	10	E	A	0.12	Destabilizing
	F	23	K	A	0.95	Destabilizing
	F	28	E	A	0.46	Destabilizing
			negative control			
YG12-2	A	52	V	A	0.35	Destabilizing
	A	61	E	A	0.28	Destabilizing
	A	91	R	A	0.44	Destabilizing
	E	25	G	A	0.49	Destabilizing
	E	31	T	A	0.21	Destabilizing
	E	55	N	A	0.63	Destabilizing
	F	5	V	A	0.32	Destabilizing
	F	31	T	A	0.21	Destabilizing
	F	60	G	A	0.38	Destabilizing
			positive control			
	A	129	Q	A	0.83	Destabilizing

	A	173	Q	A	0.86	Destabilizing
	A	180	T	A	0.66	Destabilizing
	A	189	S	A	0.26	Destabilizing
	A	193	T	A	1.10	Destabilizing
	A	195	E	A	0.53	Destabilizing
	A	235	S	A	0.45	Destabilizing
	F	23	K	A	0.95	Destabilizing
	E	49	T	A	0.69	Destabilizing

Table S6. *t*-Test results for ΔΔG differences between negative and positive control groups (YG12-1 and YG12-2).

Comparison	t-Stat	*p*-Value
YG12-1 Negative vs. YG12-1 Positive	−2.336	0.0435
YG12-2 Negative vs. YG12-2 Positive	−3.109	0.0106
